# *Schistosoma mansoni* in Family 5 Years after Safari

**DOI:** 10.3201/eid1102.040600

**Published:** 2005-02

**Authors:** Valerianna Amorosa, Daniel Kremens, Martin S. Wolfe, Timothy Flanigan, Kevin M. Cahill, Kevin Judy, Scott Kasner, Emily Blumberg

**Affiliations:** * University of Pennsylvania, Philadelphia, Pennsylvania, USA;; † George Washington University, Washington, DC;; ‡ Georgetown University, Washington, DC, USA;; § Brown University, Providence, Rhode Island, USA;; ¶ Royal College of Surgeons, Dublin, Ireland;; # New York University, New York, New York, USA;; ** Lenox Hill Hospital, New York, New York, USA

**Keywords:** letter, schistosomiasis, Schistosoma mansoni, neuroschistosomiasis, central nervous system, travel, Kenya

**To the Editor:** Each year ≈350,000 Americans travel to Africa and ≈500,000 travel to Brazil and the Far East, all schistosomiasis-endemic regions. Data from the European Network on Imported Infectious Diseases Surveillance (TropNetEurop) suggest that most schistosomiasis cases imported to Europe are acquired in Africa; 80% of new cases worldwide occur in sub-Saharan Africa (*1**,**2*). Travelers to Africa from the United States are also at high risk for infection. *Schistosoma mansoni* has the greatest impact on residents of disease-endemic areas who have high-grade infection and progressive hepatosplenic disease with portal hypertension and its manifestations. Most infected, short-term travelers sustain a low-level of fluke infestation with few symptoms, although serious complications can occur.

We report the case of a 38-year-old American man with ectopic *S. mansoni* fluke migration that led to neural schistosomiasis. His symptoms prompted us to test family members who had accompanied him on a trip to Kenya 5 years earlier. The family members had been unaware of the risk for schistosomiasis. Five years after a Kenyan safari during which the index patient visited northeastern Lake Victoria and swam one afternoon, vertigo, nausea, and nystagmus developed. The results of liver function tests were normal and peripheral blood showed no eosinophilia. Biopsy of a large cerebellar lesion noted on magnetic resonance imaging (MRI) was diagnostic, yielding multiple *S. mansoni* ova within large eosinophilic granulomas, consistent with tumoral neuroschistosomiasis. We tested for schistosomiasis 24 of 25 family members who had accompanied him to Kenya (Figure). All of the accompanying family members, except 3 women, had gone into the water. All members were well, except an 8-year-old boy, in whom granulomatous colitis had developed after the trip.

**Figure:**
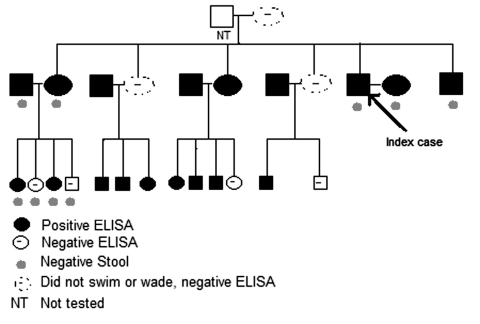
Testing for *Schistosoma mansoni* infection among family members 5 years after trip to Kenya. ELISA, enzyme-linked immunosorbent assay.

Eighteen of 25 enzyme-linked immunosorbent assays (ELISAs) were positive for *S. mansoni* infection, including that of samples from the index patient and the boy (Figure). ELISA was performed on 18 samples at the Centers for Disease Control and Prevention (CDC) and 7 samples at Focus Technologies. Both tests used the same CDC-produced antigen, the microsomal fraction of adult *S. mansoni* fluke, which has both a sensitivity and specificity for *S. mansoni* of 99%. Confirmatory immunoblots were performed at CDC on samples from 19 of the 25 ELISA-tested family members, with 1 discordant result, a positive ELISA and negative *S. mansoni* and *hematobium* immunoblots. Three of 7 ELISA-negative family members were the nonswimmers. Analyses of single stool specimens from 7 family members, including the index patient, and 1 rectal biopsy sample were negative for ova.

Because of the high positivity rate, praziquantel was prescribed for all 26 travelers. The index patient received 20 mg/kg of praziquantel twice daily for 4 days and high-dose dexamethasone with subsequent 2-month taper; his symptoms resolved over months. An MRI 8 months after treatment demonstrated minimal residual inflammation. All other family members received 20 mg/kg of praziquantel twice in a day and tolerated it without adverse events. Ten months after treatment, the boy is growing after years of an inflammatory colitis characterized by hematochezia and growth retardation. He continues to have nonbloody diarrhea and constipation.

We postulate that the mature fluke pair migrated from the mesenteric veins through Batson's vertebral-venous plexus to the cerebral veins at the cerebellar level. There, the female expelled multiple ova into the cerebellum. An ensuing vigorous granulomatous response led to posterior fossa mass effect and compression of medullary nausea centers, which resulted in the patient's nausea, vertigo, and nystagmus. Ectopic ovum migration more commonly causes neuroschistosomiasis; however, in this case, multiple ova within 1 granulomatous mass suggest fluke-pair migration rather than individual ovum migration. Neuroschistosomiasis is most commonly associated with *S. japonicum*, which has smaller ova. In the literature, we found 16 other case-patients with intracranial tumoral *S. mansoni*. Eight of the patients demonstrated cerebellar involvement, which suggests a common fluke migratory pathway (*3**–**15*). Like our patient, 6 others were not native to disease-endemic regions.

This unsuspected case of neural schistosomiasis illustrates the need for detailed inquiry into every freshwater exposure by persons who have traveled to schistosomiasis-endemic regions. Adult *Schistosoma* flukes generally survive in venules from 6 to 10 years but can survive <40 years; therefore, remote travel history is relevant. Examination of stool samples for ova has been considered the standard method of diagnosing *S. mansoni* and *S. japonicum* infection, and urine examination is used to diagnose *S. haematobium*. Multiple, fresh morning specimens are ideal. However, stool examination is not likely to be as sensitive as current immunologic assays for detecting low levels of infection. Moreover, in disease-nonendemic regions, operator variability may influence ova detection. Among 13 recorded cases of neurotumoral *S. mansoni* in which stool specimens were examined, no stool ova were found in 5 cases. In our family cohort, among the 7 ELISA-positive members who submitted stool specimens, no examinations performed at CDC demonstrated eggs (Figure).

ELISA uses a highly sensitive and specific antigen for *S. mansoni*. Because the sensitivity is less for *S. haematobium* and *S. japonicum*, subsequent species-specific immunoblots are recommended based on travel history that suggests exposure to specific species. Thus, we recommend ELISA, immunoblot if applicable, and stool or urine examination for travelers with possible exposure in disease-endemic regions. ELISA does not have the same usefulness in persons from disease-endemic regions because positivity is also consistent with earlier infection. Stool or urine examination is diagnostic in suspected immigrant case-patients.

In all cases, knowing that stool or urine examination shows ova is valuable because repeat examination at 4 to 6 weeks can be used to monitor treatment response. Because praziquantel is well tolerated and effective, empiric therapy among returning travelers after possible exposure is reasonable. However, diagnosing infection when possible and demonstrating cleared infection after therapy are more prudent approaches, particularly as praziquantel resistance emerges (*16*).

In conclusion, pretravel counseling against freshwater exposure and posttravel screening for schistosomiasis of persons with any freshwater exposure in disease-endemic regions are warranted. As illustrated, the diagnosis of schistosomiasis in a returned traveler should prompt screening for infection in fellow travelers.
